# Probabilistic assessment of drought stress vulnerability in grasslands of Xinjiang, China

**DOI:** 10.3389/fpls.2023.1143863

**Published:** 2023-03-16

**Authors:** Wanqiang Han, Jingyun Guan, Jianghua Zheng, Yujia Liu, Xifeng Ju, Liang Liu, Jianhao Li, Xurui Mao, Congren Li

**Affiliations:** ^1^ College of Geography and Remote Sensing Science, Xinjiang University, Urumqi, China; ^2^ Key Laboratory of Oasis Ecology, Xinjiang University, Urumqi, China; ^3^ College of Tourism, Xinjiang University of Finance & Economics, Urumqi, China

**Keywords:** drought stress, climate regions, grassland types, vulnerability probability, influencing factors

## Abstract

In the process of climate warming, drought has increased the vulnerability of ecosystems. Due to the extreme sensitivity of grasslands to drought, grassland drought stress vulnerability assessment has become a current issue to be addressed. First, correlation analysis was used to determine the characteristics of the normalized precipitation evapotranspiration index (SPEI) response of the grassland normalized difference vegetation index (NDVI) to multiscale drought stress (SPEI-1 ~ SPEI-24) in the study area. Then, the response of grassland vegetation to drought stress at different growth periods was modeled using conjugate function analysis. Conditional probabilities were used to explore the probability of NDVI decline to the lower percentile in grasslands under different levels of drought stress (moderate, severe and extreme drought) and to further analyze the differences in drought vulnerability across climate zones and grassland types. Finally, the main influencing factors of drought stress in grassland at different periods were identified. The results of the study showed that the spatial pattern of drought response time of grassland in Xinjiang had obvious seasonality, with an increasing trend from January to March and November to December in the nongrowing season and a decreasing trend from June to October in the growing season. August was the most vulnerable period for grassland drought stress, with the highest probability of grassland loss. When the grasslands experience a certain degree of loss, they develop strategies to mitigate the effects of drought stress, thereby decreasing the probability of falling into the lower percentile. Among them, the highest probability of drought vulnerability was found in semiarid grasslands, as well as in plains grasslands and alpine subalpine grasslands. In addition, the primary drivers of April and August were temperature, whereas for September, the most significant influencing factor was evapotranspiration. The results of the study will not only deepen our understanding of the dynamics of drought stress in grasslands under climate change but also provide a scientific basis for the management of grassland ecosystems in response to drought and the allocation of water in the future.

## 1 Introduction

Climate change is exacerbating current drought conditions (intensity, frequency and duration) around the world, and climate models predict that droughts will not abate and are likely to increase in the coming decades (Lu et al., 2019; Wu et al., 2022). Frequent drought events are often accompanied by persistent or extreme heat, leading to decreased soil moisture and increased evapotranspiration. If vegetation is affected by prolonged drought, several aspects of its growth and development (e.g., seed germination, respiration, photosynthesis, nutrient cycling) are restricted (Mohammat et al., 2013), and vegetation may brown (Li et al., 2022), decrease in biomass (Chen et al., 2022), or even die (Castellaneta et al., 2022). Vegetation response to drought is characterized by reduced vigor and photosynthesis, resulting in a decrease in the carbon sink capacity of vegetation. For more sensitive grasslands, extreme or persistent drought makes it difficult to recover from wilting or death, and dead vegetation is converted to a carbon source (Piao et al., 2019), which could have a negative impact on the global carbon cycle, as grasslands play an important role in the global vegetation carbon pool. Conversely, reductions in carbon sinks and increases in carbon sources have an impact on climate and the carbon cycle, which in turn further exacerbate drought (Reichstein et al., 2013). Vulnerability assessments of grassland drought stress under climate change have become a high-profile concern (Li et al., 2022; Zeng et al., 2022). Quantifying the response of grassland vegetation to drought stress and identifying the areas most vulnerable to drought, as well as the types of grassland that respond most strongly to drought, are therefore critical to improving our understanding of the vulnerability of grasslands to climate change and taking appropriate measures to mitigate the effects of drought.

Previous quantitative studies of drought have mostly used drought indices, which quantify accumulated moisture information and easily derive drought intensity, frequency and duration to analyze the extent of its impacts (Fang et al., 2019c). Scholars have developed different drought indices based on the research subjects and their drought characteristics. Common drought indices include the standardized precipitation index (SPI) (McKee et al., 1993) and the standardized precipitation evapotranspiration index (SPEI) (Vicente-Serrano et al., 2010), which are mainly used to study meteorological drought; the Palmer drought severity index (PDSI) (Palmer, 1965), the crop moisture index (CMI) (Palmer, 1968), and the crop water stress index (CWSI) (Jackson et al., 1988), which are mainly used to study agricultural drought; and the streamflow drought index (SDI) (Nalbantis and Tsakiris, 2009) and standardized runoff index (SRI) (Shukla and Wood, 2008), which are mainly used to study hydrological drought. Among them, the SPI is widely used because of its simple calculation and low data requirements (only precipitation data are needed) (Dutta et al., 2015; Wang et al., 2021; Yerdelen et al., 2021; Won et al., 2022), but it lacks consideration of atmospheric evaporation (evapotranspiration), which is an important factor that must be considered in the context of gradually increasing global temperatures (Fang et al., 2019c). Vegetation is sensitive to changes in water balance (precipitation minus reference evapotranspiration), especially in arid regions (Beer et al., 2007). Therefore, the inclusion of evapotranspiration as an important factor in drought and vegetation research could provide a more accurate picture of the relationship between drought and vegetation conditions.

The normalized difference vegetation index (NDVI) is used to describe and conduct terrestrial vegetation condition assessments due to its ease of accessibility at different spatial and temporal resolutions and its advantage of eliminating noise caused by solar angle, topographic illumination, cloud cover and atmospheric conditions, and the NDVI is widely used in studies related to grasslands, forests and agricultural lands (Estel et al., 2015; Kowalski et al., 2022; Wang et al., 2022; Xun et al., 2022). Previously, the response of vegetation to drought was expressed as a correlation between the NDVI and drought index, demonstrating that there is a good correlation between the NDVI, which represents vegetation, and a drought index, which represents moisture conditions, and that there is a significant effect of drought occurrence on vegetation. Among them, GIMMS NDVI has the disadvantage of low resolution and easy saturation compared with other satellite sensors (Sentinel, Landsat and SPOT, etc.) and vegetation indices (EVI, DVI and SAVI, etc.). However, when considering the effects of arid vegetation under climate change, data with long time series are needed to support accuracy, and GIMMS performs best in the AVHRR-derived NDVI dataset for temporal changes. Therefore, the GIMMS NDVI is favored by scholars.

Studies on vegetation response to drought have been conducted globally (Ji and Peters, 2003; Xu et al., 2016). Vegetation in arid and semiarid regions with longer sunshine duration is more susceptible to drought (Gouveia et al., 2017) than that in humid regions with a positive water balance (Zhang et al., 2017). The drivers of vegetation drought are also of scientific interest to scholars, and the main influences may vary with the growing season. In addition, the topography (elevation, slope and slope direction) affects the aridity pattern of the vegetation, as it directly influences the temperature and solar radiation in the region. It is noteworthy that grasslands respond more strongly to drought than do other vegetation types, and there may be differences in responses between grassland types (Huang et al., 2021; Bu et al., 2022). The response of grasslands to drought also changes in response to changing climatic conditions (Wellstein et al., 2017; Liu et al., 2019; Zhang et al., 2020). Regarding vegetation, there is a need to pay more attention to the growing season (seasonal time) of grassland vegetation (Zhang and Liu, 2014; Mina et al., 2016), as this is the most severe phase of vegetation drought stress (Ji and Peters, 2003). The grassland vegetation response to drought is not instantaneous but is caused by a cumulative water deficit over time, as reflected in the response time of vegetation to drought (lag effect) (Zhao et al., 2020; Zhang et al., 2022). When faced with prolonged drought, grasses also adopt different strategies to adapt to water scarcity, such as closing stomata (Li and Liu, 2022), evolving stronger roots, and even having drought memory (Yao et al., 2022). Among them, photosynthesis of vegetation is the most important link, which regulates its own growth and development to adapt to drought. In addition, vegetation can maintain its health and growth by regulating stomatal conductance when it faces water deficits or water surpluses. Recently, the copula approach was used to determine the dependence of vegetation on water, providing scholars with an effective tool to address the vulnerability of vegetation at the onset of drought (Bento et al., 2018; Fang et al., 2019c). Xinjiang is a typical arid and semiarid region, and as the core economic zone of the Silk Road in China, it has experienced 26 severe and above droughts between only 1961 and 2000, and the increased warming and reduced precipitation have increased the local evapotranspiration (ET_0_), intensifying local drought and vegetation. The ecosystem is vulnerable to the effects of drought and heat (Yao et al., 2018; Yao et al., 2019). Grasslands are widely distributed in Xinjiang, accounting for 86% of the vegetation, and play an important role in climate regulation, soil and water conservation, wind and sand control, biodiversity conservation, and carbon cycling (Du et al., 2022). Therefore, it is necessary to assess the drought vulnerability of grasslands in Xinjiang and to elucidate the heterogeneity among different climatic regions and grassland types. However, previous studies on drought in Xinjiang have been limited to deterministic assessments of drought impacts, focusing on vegetation growth and drought conditions or drought response rates. There are still gaps in assessing grassland losses under different levels of drought stress. In addition, there are knowledge gaps in analyzing the probabilistic response of grassland loss under drought stress from the perspective of different climatic zones and grassland types.

To this end, the aim of this study was to further quantify the dynamic response of grasslands to drought stress. The objectives were to (1) determine the scale of grassland NDVI (grassland growth state) response to changes in the SPEI (multiscale drought stress) during different growth periods through correlation analysis; (2) clarify the probabilistic differences in response to drought stress among different climatic regions and grassland types after estimating the probability of occurrence of grassland below normal status under three drought scenarios (moderate, severe and extreme) based on the established copula conditional probability distributions; and (3) analyze the main influencing factors of drought stress in grassland at different periods. The results of the study can improve our understanding of grassland vulnerability under climate change and help decision-makers understand vulnerable grassland areas under different drought scenarios from a probabilistic perspective, which is important for reducing the pressure on grasslands in arid regions.

## 2 Material and methods

### 2.1 Study area

**Figure 1:**
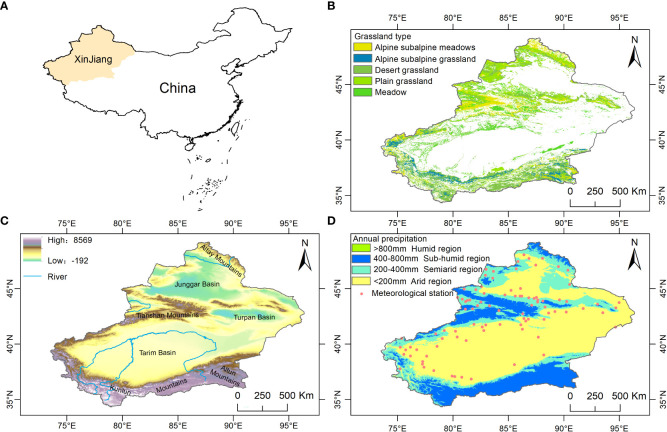
**(A)** Location map of Xinjiang, China, **(B)** grassland types, **(C)** topographic attributes, and **(D)** classification of climatic regions.

Xinjiang is located in northwestern China (73°40′-96°23′E, 34°25′-49°10′N), deep in the Eurasian continent, accounting for one-sixth of the national territory, and it is the largest provincial administrative division in China (**Figure 1**). The geomorphology is characterized by a closed system of “three mountains sandwiched by two basins adjacent to each other” (**Figure 1C**). The two basins are the Junggar Basin in the north and the Tarim Basin in the south, and the mountains are the Altai Mountain range in the north, the Kunlun Mountains in the south, and the Tianshan Mountains, which divide Xinjiang into the north and south (Guli et al., 2015). Xinjiang is a typical arid region with 2550-3500 hours of annual sunshine per year (He et al., 2021), an annual precipitation of 100-200 mm and a potential evaporation of 2000-3400 mm per year (Liu et al., 2018). Grassland is distributed mainly at the edge of mountains and basins, with the Altai, Tianshan and Kunlun Mountains being the most obvious (**Figure 1B**).

### 2.2 Normalized difference vegetation index and grassland type

The NDVI data were from the NASA Ames Ecological Forecasting Lab (https://ecocast.arc.nasa.gov/) GIMMS-NDVI for the period 1982-2015 with a spatial and temporal resolution of 1/12° (~8 km) and 15 d and the NASA Distributed Active Archive Center for Terrestrial Processes (https://pdaac.usgs.gov/) for the period 2000-2020 for MODIS-NDVI with a spatial and temporal resolution of 0.05° and 1 month. The data were adopted due to the good linear correlation between the two data sources (Li et al., 2017). The GIMMS-NDVI was first synthesized into maximums and then into a monthly scale NDVI, thus reducing the effects of atmospheric and aerosol scattering (HOLBEN, 1986). Finally, MODIS-NDVI was resampled to the same resolution as GIMMS-NDVI, and a pixel one-dimensional linear regression model was developed using overlapping data from 2000-2015 (Xu et al., 2016), extending the GIMMS-NDVI monthly data from 1982-2015 to 1982-2020 (Guan et al., 2021). Grassland type data were from the Global Land Cover Data Product 2000 (GLC2000) of the Institute for Space Applications of the Joint Research Centre of the European Union. There are 22 land use types (https://forobs.jrc.ec.europa.eu/products/glc2000/legend.php) with a spatial resolution of 1 km. The main types of grasslands in Xinjiang are alpine and subalpine meadows, plain grasslands, desert grasslands, meadows, and alpine and subalpine grasslands (**Figure 1B**), and they were resampled to 1/12° to be in agreement with the NDVI.

### 2.3 Standardized precipitation evapotranspiration index

From 1961 to 2020, the National Meteorological Information Center of the China Meteorological Administration (http://www.nmic.gov.cn/) provided raw records of daily precipitation and temperature from 105 weather stations in Xinjiang. Only 92 of these meteorological stations had continuous daily records for the same period (**Figure 1B**), and the SPEI was calculated in the R language and interpolated to 1/12° by the inverse distance weighting method.

**Figure 2:**
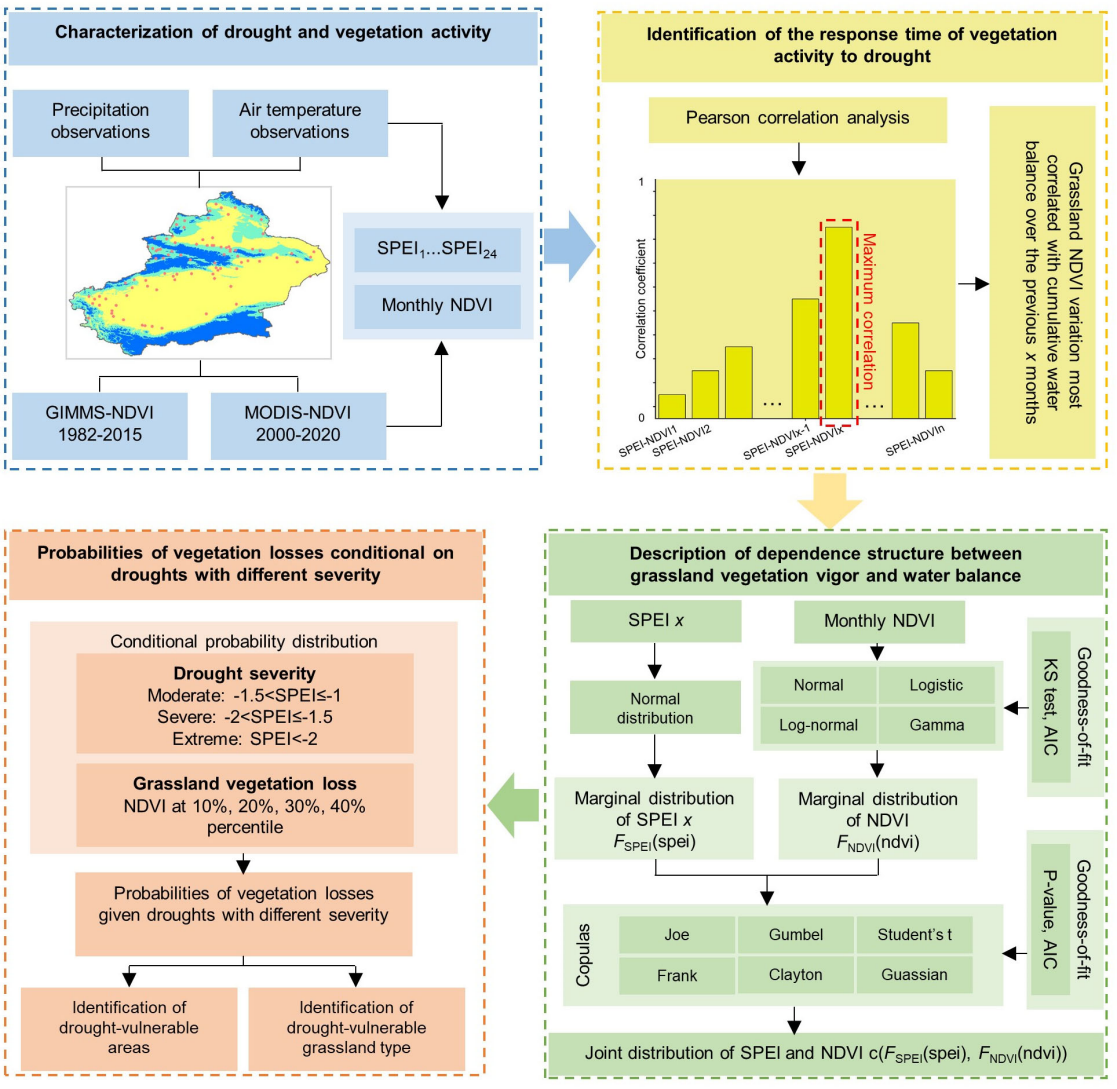
Copula probability model for the response of Xinjiang grassland to drought stress.

**Figure 3:**
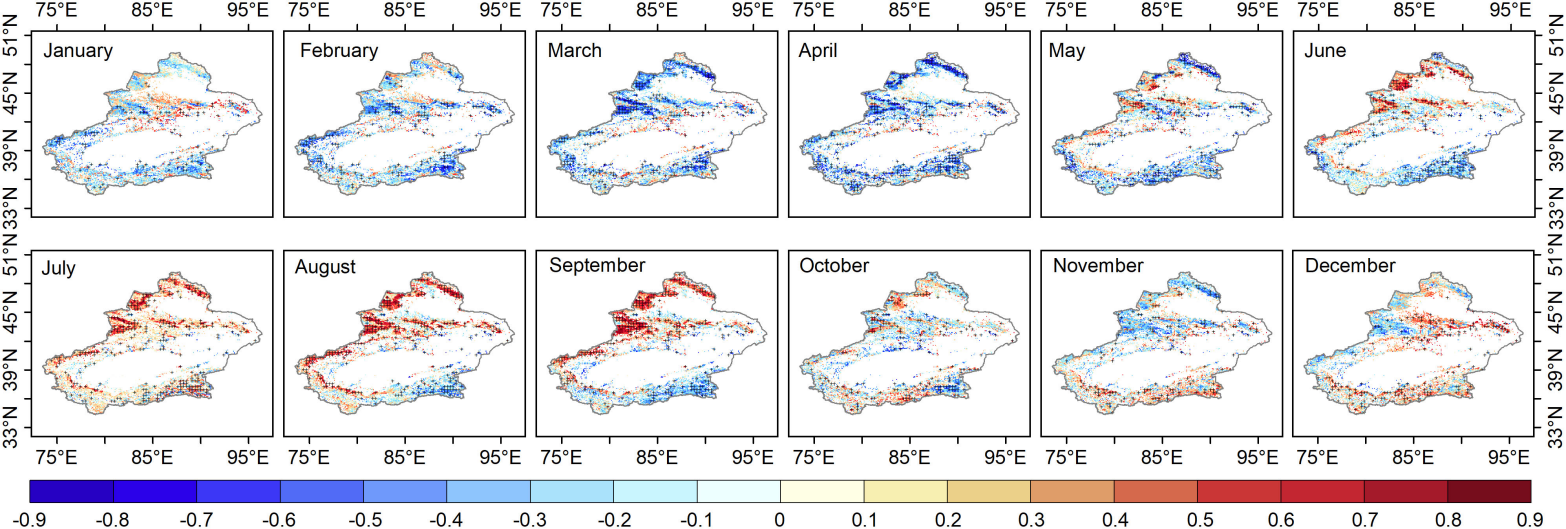
Pixels with maximum correlation between 1982-2020 grassland NDVI and SPEI1-24 pixel by pixel; (p<0.1) are covered by a black cross.

Precipitation minus potential evapotranspiration is defined as the water balance *(D)*:


(1) $$D=P-ET_{0}$$

where *P* and *ET*_0_ represent the monthly precipitation and potential evaporation, respectively. *ET*_0_ was calculated using the Thornthwaite equation (Thornthwaite, 1948) because it is easy to calculate using less data (only the monthly mean temperature and latitude are required) and works well (Fang et al., 2018). The SPEI timescales can reflect different droughts (meteorology, agriculture, climate change, etc.). Dry and wet states were classified according to the SPEI classification criteria (**Table 1**). In this study, the SPEI was calculated for multiple time scales (1-24 months) (Vicente-Serrano et al., 2013). The grass reaction time was depicted as the time scale of the highest SPEI-NDVI correlation in **Figures 2**, **3** (Zhang et al., 2022).

**Table 1:** SPEI wet and dry classification criteria.

Wet and dry level	Extremely Wet	Heavy wet	Moderately wet	Normal state	Moderate drought	Severe drought	Extreme drought
SPEI value	SPEI > 2	1.5 < SPEI ≤2	1 < SPEI ≤1.5	−1 < SPEI ≤1	−1.5 < SPEI ≤−1	−2 < SPEI≤ −1.5	SPEI≤ −2

### 2.4 Methods

The response to drought stress in the Xinjiang grassland was quantified using a copula probabilistic model (**Figure 2**).

The copula method was used to describe different drought conditions (different SPEI thresholds) and grassland status (NDVI values) as a joint distribution to describe the dependencies between drought and grassland. Subsequently, the probability of the grassland NDVI declining to the lower percentile under moderate, severe and extreme drought conditions was calculated based on the joint distribution.

#### 2.4.1 Joint probability distribution

The dependence of grassland NDVI on different drought levels was established by the copula function. Assuming that the SPEI and NDVI are represented by *S* and *N*, respectively, and using the copula function *(C)*, the joint distribution *F_s_* (*s*,*F_N_* (*n* is expressed as follows:


(2) $$P(S⩽s,N⩽n)=F_{SN}(s,n)=C\left[F_{S}(s),F_{N}(n)\right]$$

where *F_s_* (*s* and *F_N_* (*n* are the cumulative distribution functions (CDFs) of the SPEI and NDVI, respectively.

In this study, the normal distribution was employed for the SPEI’s marginal distribution. In previous studies, it was difficult to achieve a consistent marginal distribution of the NDVI (Wen et al., 2012; Zhao et al., 2019). Therefore, the Kolmogorov-Smirnov (K-S) test and the Akaike information criterion (AIC) were used to select the optimal marginal distribution from the logarithmic, gamma, log-normal and normal distributions (Yu et al., 2018). Thus, to model the response of grassland NDVI to drought stress, six copulas (Joe, Frank, Gumbel, Clayton, Gaussian and student) were selected, in which Gaussian and student were able to effectively resolve the negative correlation that emerged in April (Grimaldi et al., 2016; Zhou et al., 2018). Then, the optimal connection function was selected using the maximum likelihood (MLE) method and the goodness-of-fit test, and the best copula model was screened using Cramérévon (Fang et al., 2019a).

#### 2.4.2 Condition distribution of the SPEI and NDVI

Statistically, when drought occurs, the probability of grassland NDVI declining to lower percentiles is denoted as conditional probability *P*(*S*≦̸*s*,*N*≦̸*n*. The joint distribution and drought classification can be used to derive conditional probability equations and estimate the grassland NDVI under different drought levels. In this study, we considered scenarios in which grassland normalization values were lower than the prescribed percentage in different degrees of drought (including moderate drought (-1 < SPEI ≤ -1.5), severe drought (-1.5 < SPEI ≤ -2), and extreme drought (SPEI < -2), as shown in Eqs. (3), (4) and (5), respectively:


(3) $$P(N<n|-1.5<S⩽-1)=\frac{P(-1.5<S⩽-1,N<n)}{P(-1.5<S⩽-1)}=\frac{F_{SN}(-1,n)-F_{SN}(-1.5,n)}{F_{S}(-1)-F_{S}(-1.5)}$$

(4) $$P(N<n|-2<S⩽-1.5)=\frac{P(-2<S⩽-1.5N<n)}{P(-2<S⩽-1.5)}=\frac{F_{SN}(-1.5,n)-F_{SN}(-2,n)}{F_{S}(-1.5)-F_{S}(-2)}$$

(5) $$P(N<n|S⩽-2)=\frac{P(S⩽-2,N<n)}{P(S⩽-2)}=\frac{F_{SN}(-2,n)}{F_{S}(-2)}$$

Eqs. (2)-(5) quantify the response of grassland NDVI to changes in the SPEI. Grassland NDVIs below the 40th, 30th, 20th, and 10th percentiles for moderate, severe, and extreme drought scenarios were considered in the study.

It is worth noting that predicting the vulnerability of grasslands to drought is more meaningful given the specific values of the drought index (P (NDVI < ndvi | SPEI = spei)). In this study, the probability density function (PDF) formula was as follows (Mazdiyasni et al., 2017):


(6) $$f_{N|S}\left(n|s\right)=c\left[f_{S}\left(s\right),f_{N}\left(n\right)\right]\cdot f_{N}\left(n\right)$$

where c represents the chosen copula function, and *fs* and *f_N_* represent the PDF versions of the SPEI and NDVI, respectively. By calculating the PDF (S = *s*) under specific drought conditions according to Eq. (6), the cumulative probability of the grassland NDVI declining to a specific value (N<n) was derived and denoted as P(N<n∣S=*s*.

## 3 Results

### 3.1 Spatial pattern of the SPEI-NDVI correlation

The correlation between the grassland NDVI and SPEI at different time scales (1-24 months) can determine the response time of grassland to moisture changes. There is a time lag effect in the vegetation response to moisture change, so the response time of this study can be defined as the scale with the most considerable SPEI-NDVI correlation. The largest correlation coefficients between grassland NDVI and SPEI_1-24_ per pixel occurred over 12 months (**Figure 3**). At this point, the SPEI-NDVI correlation showed a distinct spatial pattern, and the year was divided into the growing period (April-October), spring (April-May), summer (June-August), autumn (September-October), and nongrowing season (November-December and January-March).

Our study showed that in contrast to the growth season, the spatial variability of the SPEI-NDVI correlations was lower in the grasslands of Xinjiang. The SPEI-NDVI correlations showed obvious spatial heterogeneity during the growing season, and the correlations in the north were significantly stronger than those in the south. The spatial differences were obvious throughout the growing season (May-September) but not in April or October. The regional boundaries of high and low SPEI-NDVI correlations from May-September were basically consistent with the boundary of the “three mountains and two basins” in Xinjiang. As shown in **Figure 1D**, most of the semi-humid regions (annual precipitation greater than 400 mm and less than 800 mm) and semiarid regions (annual precipitation greater than 200 mm and less than 400 mm) were in northern Xinjiang. The correlation coefficients were higher in the Altay Mountains and Tian Shan (semi-humid and semiarid regions) in northern Xinjiang than in southern Xinjiang (semiarid regions).

### 3.2 Response time of grassland to water activity

**Figure 4:**
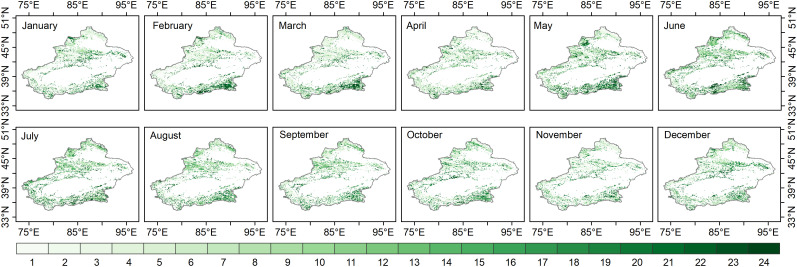
The response time of grassland to water activity (the SPEI time scale with the highest correlation between the SPEI and NDVI on each image element).

The response time of grassland to water activity (multiscale drought stress) in this study was defined as the maximum scale of the SPEI-NDVI correlation on each image element, while representing the lag of grassland response to water. In the nongrowing season, the response time of the Xinjiang grassland was different. The grassland response time increased in January-March and November-December compared to the SPEI-NDVI correlation in different months (**Figure 4**). In the growing season, the response time of grassland in most areas of Xinjiang, especially the mountainous areas at higher elevations, to moisture variability began to increase in April-May, with the longest response time in May and a downward trend in June-October.

The above spatial patterns of responses at different time scales indicated that the response time of grassland communities in the semi-humid regions of Xinjiang to moisture changes was longer than that in the arid and semiarid regions during the growing and nongrowing seasons. While the nongrowing seasons of January-March and November-December generally had an increasing trend in grassland response times, the growing seasons of June-October had a decreasing trend in response times.

### 3.3 Probability of grassland decline under different drought stresses

**Figure 5:**
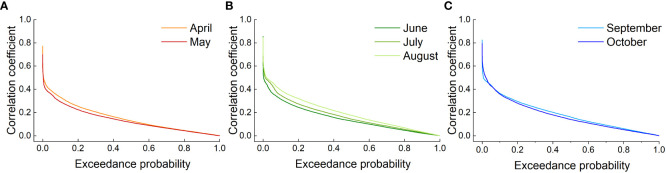
The SPEI-NDVI correlation coefficient exceeded the probability curves for Xinjiang grasslands in the growing seasons in **(A)** spring, **(B)** summer and **(C)** autumn.

Grassland activity during the growing season was higher than that during the nongrowing season, which largely determined the grassland biodiversity, biomass or yield. However, assessing the impact of drought in all 12 months of the year is not easy. A high correlation between the NDVI and SPEI would better reflect the grassland response to moisture changes and identify the most responsive months of the grassland drought-affected growing season (Zhong et al., 2021). This would represent the worst scenario for vegetation loss at different degrees of drought. Therefore, this study used the month with the highest correlation coefficient for the corresponding season to analyze the response of grassland to drought. The transcendental probability of the SPEI-NDVI correlation was calculated for all grassland image elements in the study area to determine the month that best represented the corresponding season (**Figure 5**). **Figure 5A** shows that the SPEI-NDVI correlation in April had a higher probability of exceeding a given value compared to the SPEI-NDVI correlation in May, so April was the representative month of spring. The same was true for **Figure 5B** in summer and **Figure 5C** in autumn of the growing season, so August and September were chosen as the representative months of the corresponding seasons, respectively.

Using Eqs. (2)-(5), the probabilities of conditions causing grassland deterioration in three drought scenarios, moderate drought, severe drought and extreme drought (i.e., -1.5 < SPEI ≤ -1, -2 < SPEI ≤ -1.5 and SPEI ≤ -2, respectively), were calculated. In addition, the probability of grassland deterioration status due to different droughts was comprehensively estimated to identify drought-prone areas. This study focused on grassland NDVI below the 40th, 30th, 20th and 10th percentiles. A lower NDVI percentile indicates severe grassland loss and damage to an ecosystem.

**Figure 6:**
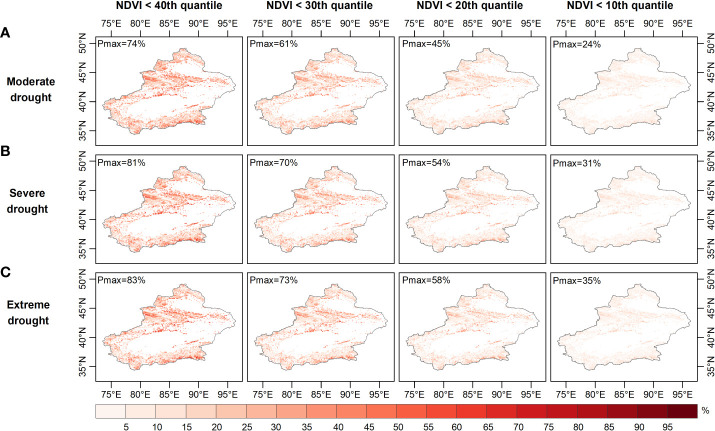
Probability of grassland NDVI deterioration in spring under **(A)** moderate, **(B)** severe, and **(C)** ex-treme drought conditions.

**Figure 7:**
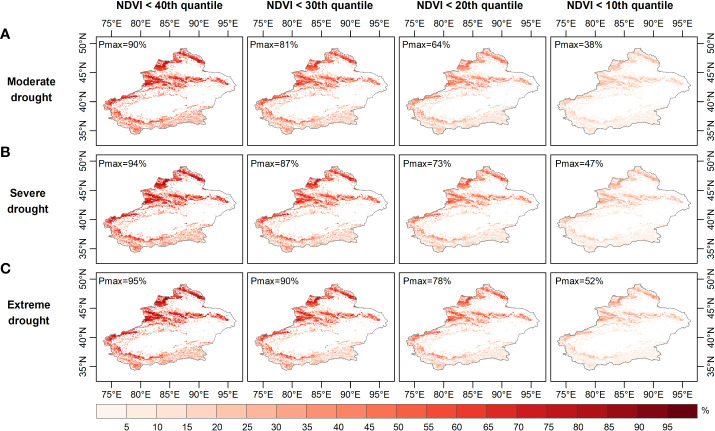
Probability of grassland NDVI deterioration in summer under **(A)** moderate, **(B)** severe, and **(C)** ex-treme drought conditions.

**Figure 8:**
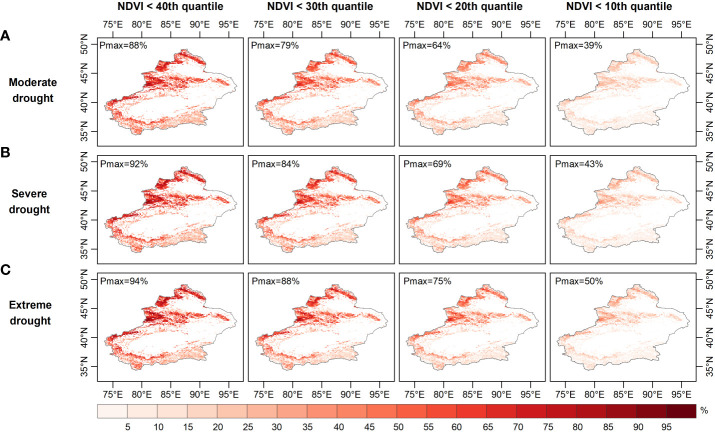
Probability of grassland NDVI deterioration in autumn under **(A)** moderate, **(B)** severe, and **(C)** ex-treme drought conditions.

**Figures 6**–**8** show the probability of vegetation condition deterioration under the three drought scenarios during the growing season in April, August and September. In April, the probability of the NDVI falling below 40 percent varied little, as moderate drought progressed to severe or even extreme drought (**Figures 6**–**8**). Conversely, the probability of the NDVI falling below 40 percent tended to increase in August and September with increasing drought levels. When considering a grassland NDVI below the 30th, 20th, and 10th percentiles in April, August, and September, respectively, it was further confirmed that August and September showed a positive response in the probability of deterioration of grassland NDVI with increasing drought, while the response was not significant in April. This result also shows the higher probability of a grassland NDVI decline during the growing season in areas with more severe water deficits when the grassland vegetation is at a lower percentage, consistent with the results of other regional studies (Liu et al., 2016; Won et al., 2021; Yuan et al., 2022). Furthermore, region-wide, the probabilities of moderate, severe, and extreme drought scenarios below the 40th percentile were 28.7%, 27.5%, and 27.0% in April; 47.6%, 48.7%, and 49.2% in August; and 47.6%, 48.6%, and 49.1% in September, respectively (**Figures 6**-**8**).

When grassland vegetation conditions deteriorated to the lower percentile (NDVI dropped below the 30th and 20th percentiles), the mean probability difference in vegetation conditions increased to -0.31% and -0.15% (NDVI ≤ the 30th percentile) and -0.15% and -0.42% (NDVI ≤ the 20th percentile), respectively, for the three drought scenarios in April; additionally, the average probability variance increased to 0.59%, 0.67%, 1.4%, and 1.6% in August and 0.58%, 0.67%, 1.4%, and 1.6% in September. When considering the worst grassland status (NDVI ≤ the 10th percentile), the average probability of the Xinjiang grassland being in the three drought scenarios in April was 6.5%, 6.4% and 6.3%, respectively, and in August it was 13.6%, 14.9% and 15.4%, respectively. Moreover, when extreme drought occurred in April, the probability of the grassland NDVI decreasing to less than the 10th percentile was less than that during severe and moderate drought. In contrast, the extremes in August and September both resulted in the highest probability of a decrease in grassland NDVI below the 10th percentile. When considering conditions where the grassland NDVI was below a specific percentile (40th, 30th, 20th, 10th), the difference in the probability of the NDVI for grassland increased from -1.7%, -1.1% and -0.6% to -0.2%, respectively, under extreme and moderate drought conditions in April. The difference in the probability of grassland NDVI being between extreme and moderate drought scenarios increased from 1.5%, 2.0% to 2.3% in August and then decreased to 1.8%, with a similar situation in September. Thus, probabilistic analysis quantitatively demonstrated that grasslands were more likely to experience a significant decline with increasing drought severity and would deteriorate to a point where they made appropriate adaptation strategies to mitigate the effects of drought (Wilcox et al., 2020). August was the period when grassland vegetation was most responsive.

Areas vulnerable to water scarcity have long been a concern for policy-makers. Probabilistic analysis of drought-prone and vulnerable grassland areas may contribute to effective drought prevention and mitigation. For this purpose, the probability of grassland loss (NDVI less than the 40th, 30th, 20th and 10th percentiles) was calculated based on the image pixel scale. The probability of grassland NDVI decline was relatively high in the northern (especially the Tian Shan) and southern (the Kunlun) regions under moderate drought conditions (**Figure 6A**). The drought-prone areas identified for these occurrences of moderate, moderate and severe drought were essentially the same in April (**Figures 6B, C**). As **Figure 7** and **Figure 8** show, the drought-prone areas in August and September were generally the same as those in April under the three drought scenarios, and the grasses in these areas were more likely to fall into the lower percentile. The difference is that the probability of decline in grasslands in August and September in northern Xinjiang was more sensitive than that in southern Xinjiang, where the Tian Shan and Altai Mountains were drought-sensitive areas, and the Tian Shan was the area most sensitive to water scarcity. Therefore, the Tianshan Mountain grasslands in northern Xinjiang and northern Xinjiang were poor drought-tolerant regions during the growing season. In a previous study, a vegetation response assessment of the Tianshan Mountain grasslands in northern Xinjiang (Li et al., 2021) showed that the Tianshan Mountain grasslands were highly sensitive to water deficits, consistent with the drought-prone areas identified in this study. In addition, the drought-prone areas identified in this section showed a high correlation of the SPEI-NDVI in **Figure 3**, demonstrating that areas with high SPEI-NDVI correlation coefficients were susceptible to drought stress.

### 3.4 Probability of loss under drought stress in different climatic regions and grassland types

**Figure 9:**
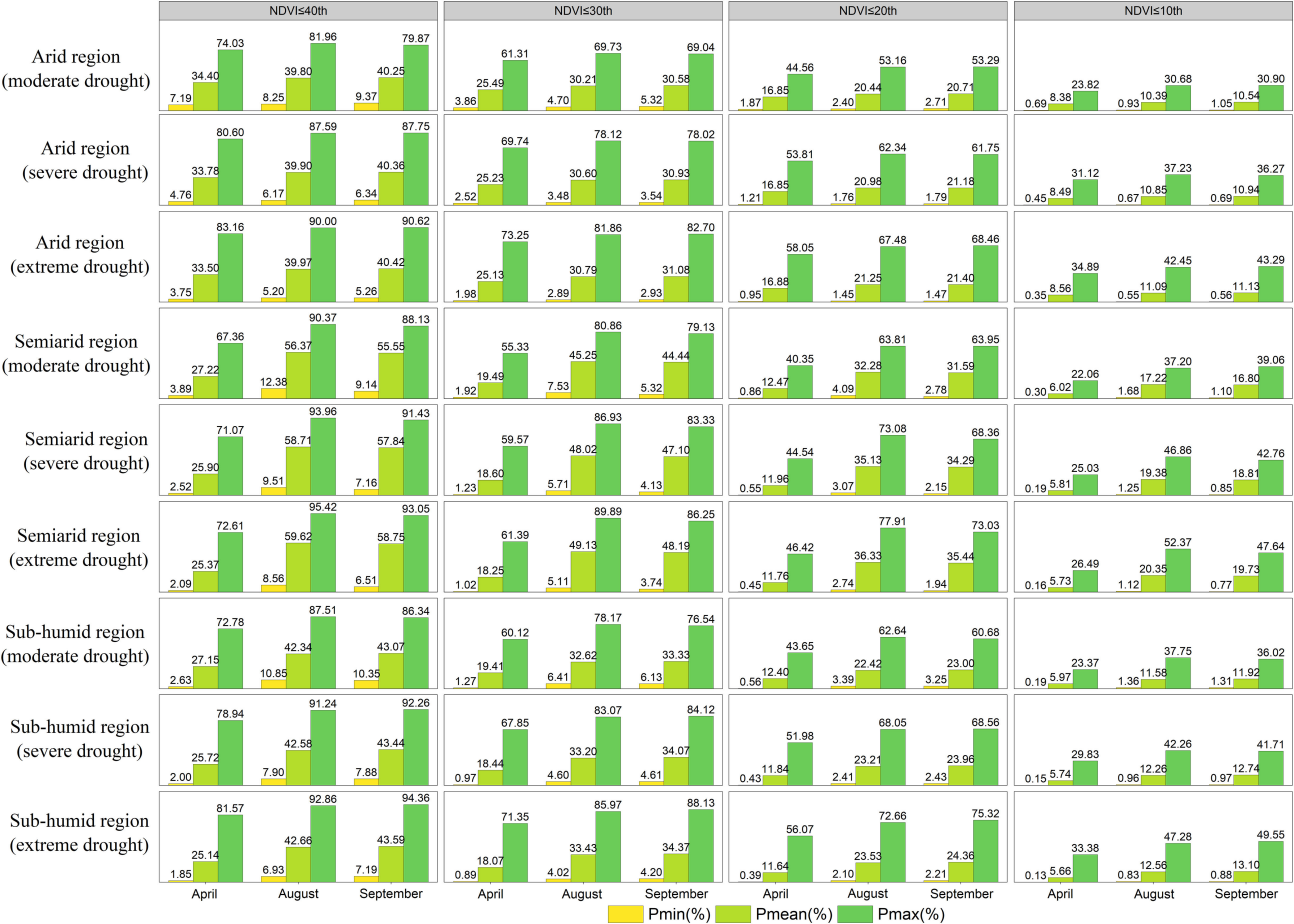
The grassland NDVI under drought stress (moderate, severe, extreme drought) in different climatic zones (arid, arid and subhumid regions) in April, August and September was lower than the lowest, average and maximum probabilities of the 40th, 30th, 20th and 10th percentiles.

Grassland degradation under drought stress in different climatic regions (arid, semiarid and semi-humid regions) was assessed from the perspective of climatic regions (**Figure 9**). The mean probability of grassland declining to a lower percentile when different degrees of drought occurred in April was highest for grassland in arid regions, while the mean probability was highest for semiarid regions in both August and September. The difference between the probability of grasses in the dry areas declining to the 30th, 20th and 10th percentiles under moderate, severe and extreme drought and the average probability in Xinjiang in April was 4.7%, 5.3% and 5.5%, 3.5%, 3.9% and 4.1% and 1.9%, 2.1% and 2.3%, respectively. The difference between the probability of semiarid regions and the average probability of Xinjiang in August was 8.0%, 9.3% and 9.8%, 6.2%, 7.5% and 8.0%, and 3.6%, 4.5%% and 4.9%, respectively. In September, the differences were 7.1%, 8.3% and 8.8%, 5.5%, 6.7% and 7.2%, and 3.2%, 4.0% and 4.3%, respectively. The semiarid regions in August and September (summer) were more prone to drought than they were in April (spring), including in Tianshan, Altay and parts of Kunlun in northern and southern Xinjiang, which were more drought-sensitive.

**Figure 10:**
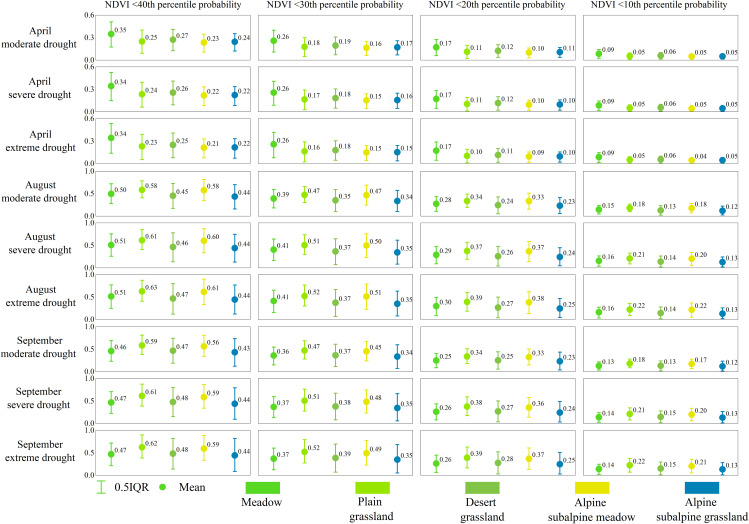
The grassland NDVI under drought stress (moderate, severe, extreme drought) in different climatic zones (meadow, plain grassland, desert grassland, alpine subalpine meadow and alpine subalpine grassland) in April, August and September was lower than the average probabilities of the 40th, 30th, 20th and 10th percentiles, respectively.

The probability of decline to the lower percentile under drought stress was assessed for different grassland types (meadow, plain grassland, desert grassland, alpine subalpine meadow and alpine subalpine grassland) (**Figure 10**). When drought occurs, grasslands are subjected to different degrees of drought stress, and the probability that the NDVI will drop to a lower percentile varies depending on the type of grassland. Among them, the probability of meadows falling to a lower percentile in April was the highest, and the probability of alpine subalpine meadows falling to a lower percentile was the lowest, with the order of probability being meadows > desert grasslands > plains grasslands > alpine subalpine grasslands > alpine subalpine meadows. The highest probability of decline to the lower percentile was found in August and September for plain grasslands, and the lowest probability was found for alpine subalpine grasslands, with the order of probability being plain grasslands > alpine subalpine meadows > desert grasslands > meadows > alpine subalpine grasslands.

### 3.5 Climate influencing factors of different seasons in grassland

**Figure 11:**
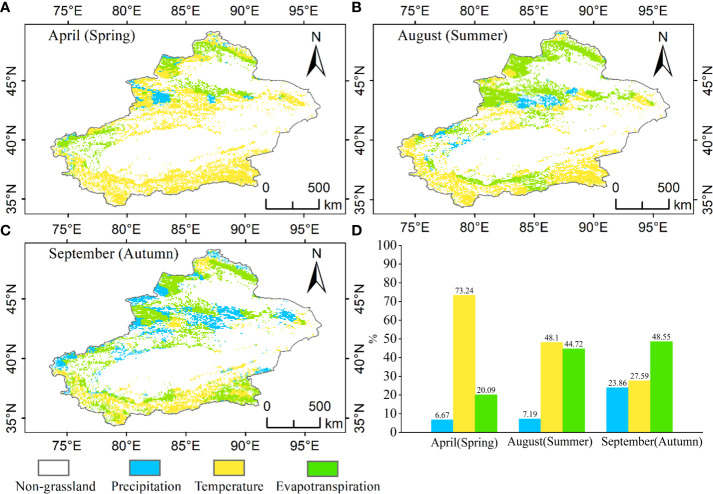
Distribution of climate influencing factors in Xinjiang grasslands, **(A)** distribution of impact factors in spring (April), **(B)** distribution of impact factors in summer (August), **(C)** distribution of impact factors in September (autumn), **(D)** statistics on the distribution of climate influencing factors in different growing periods.

The main climatic factors affected grasslands change with the period of growth (**Figure 11**). The main climatic factor affecting the growth of grasslands in April in spring was temperature, and these sites were distributed mainly in the Tianshan, Kunlun and Altun Mountains, accounting for 73.24% of the entire grassland, 6.67% of the area affected by precipitation and 20.09% of the area affected by evapotranspiration. The main climatic factor affecting the growth of grassland in summer August was again temperature, and the sites were mainly distributed in the Kunlun and Altun Mountains, accounting for 48.10% of the entire grassland, 7.19% of the area affected by precipitation and 44.72% of the area affected by evapotranspiration. The main climatic factor affecting the growth of grassland in September in autumn was evapotranspiration, and the sites were mainly distributed mainly in the Kunlun and Altun Mountains, accounting for 48.55% of the whole grassland, 23.86% of the area affected by precipitation and 27.59% of the area affected by evapotranspiration.

### 3.6 Reliability validation of the copula model

**Figure 12:**
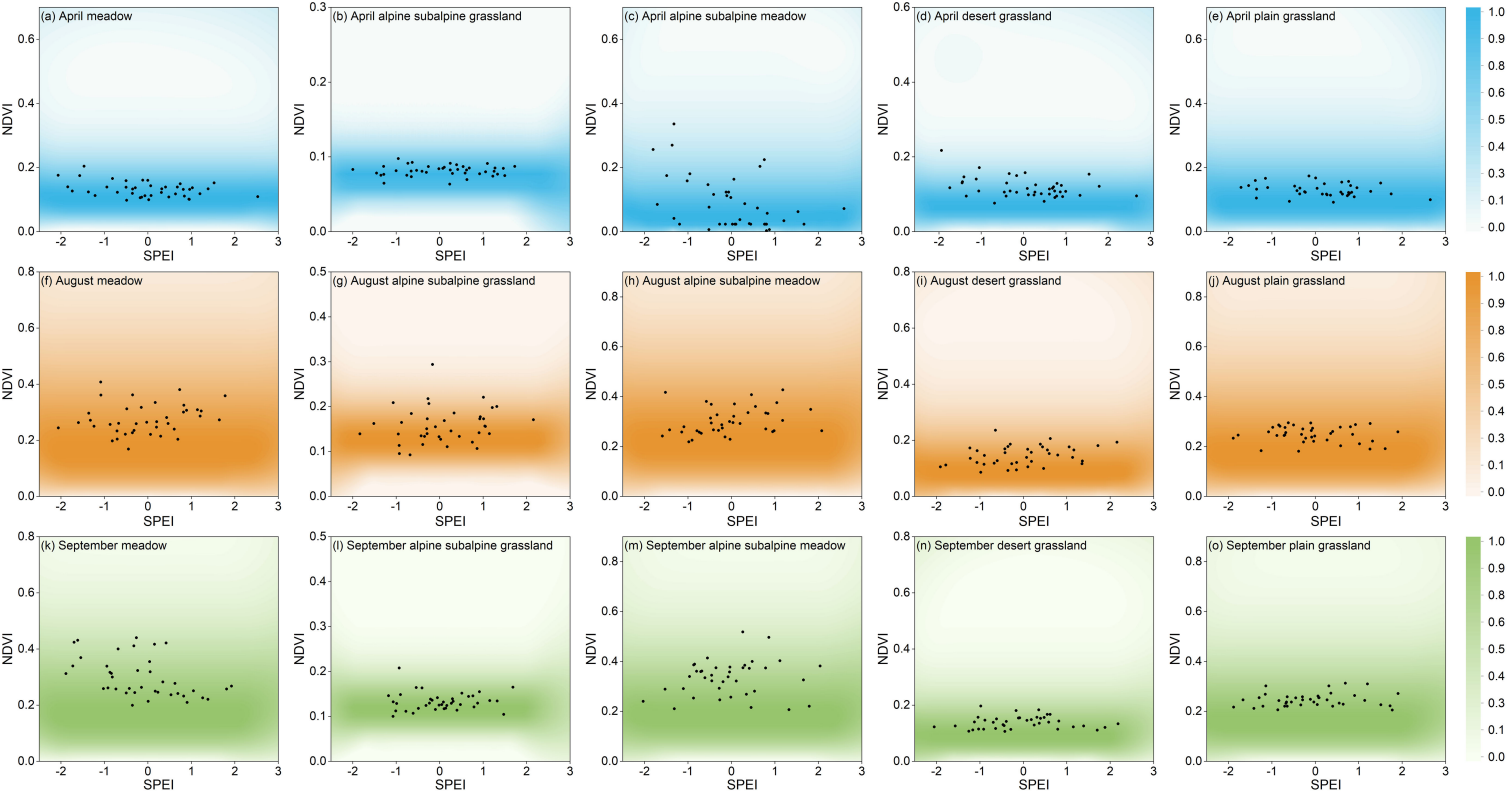
Comparing the grassland NDVI (normalized between 0-100%) with the paired NDVI-SPEI (black points), **(A–O)** are five randomly selected pixel points from five grassland types in April, August and September, respectively.

By comparing the NDVI conditional distribution with the corresponding NDVI-SPEI paired observations from 1982-2020, the model was validated for the probability derivation of complex structures under drought conditions and the identification of drought-prone areas. The NDVI distribution of the five grassland types in Xinjiang in spring, summer and autumn (April, August and September) was simulated using the conditional distribution method of Eq. (6). Of all pixel sites in Xinjiang, five were randomly selected for model validation: meadow, alpine and subalpine grassland, alpine and subalpine meadow, desert grassland and plain grassland. **Figures 12A–I**, **K–O** show the five grassland types in April, August, and September, respectively, with color shadows representing the PDF values for a given SPEI value, and the PDF values are standardized. Most of the paired observations were in the high-density area of the conditional probability function. The results showed that the probabilistic model of drought stress in Xinjiang grassland based on copula was reliable.

## 4 Discussion

### 4.1 Correlation and response time of grassland with drought

The results of previous related studies showed that the SPEI-NDVI correlations reflected different correlations and spatial characteristics depending on the vegetation growth period (Zhao et al., 2018; Zhou et al., 2018; Li et al., 2022). The NDVI was positively correlated with the SPEI in grassland communities in most areas of Xinjiang from November to December. Xinjiang’s location in the heartland of the Asian continent has a significant impact on its climatic conditions. The region is primarily influenced by westerly circulation, which results in low moisture conditions. As a result, the growth of grasslands is limited due to the scarcity of water. (Yao et al., 2020). The negative SPEI-NDVI correlation occurred mainly at some high latitudes (mainly the Altai Mountains, the Tian Shan and the Kunlun Mountains). Lower temperatures in late autumn (November-December) and winter (January-March) reduced the enzyme activities required for photosynthesis, respiration, and transpiration and reduced the water demand for grassland vegetation growth; additionally, they were the leading causes of grassland biomass decline in the high latitudes of Xinjiang (Neuner et al., 2020). From March to May, despite the low precipitation, the rising temperatures increased the soil moisture as a result of melting snow and ice in the mountains, allowing grasslands in northern and southern Xinjiang and central Tian Shan to recover (Duan et al., 2021). Therefore, when snow and glaciers provided additional soil moisture, grassland recovery in spring was less dependent on precipitation. The same result was reported by Yao et al. (2019), who, in their study on the influence of hydroclimatic variables on the NDVI in Xinjiang, noted that soil moisture varied more significantly in response to vegetation dynamics, reflecting that the weaker NDVI-SPEI correlation in spring was due to the availability of sufficient snow or glaciers for soil moisture replenishment (Yao et al., 2019).

**Figure 13:**
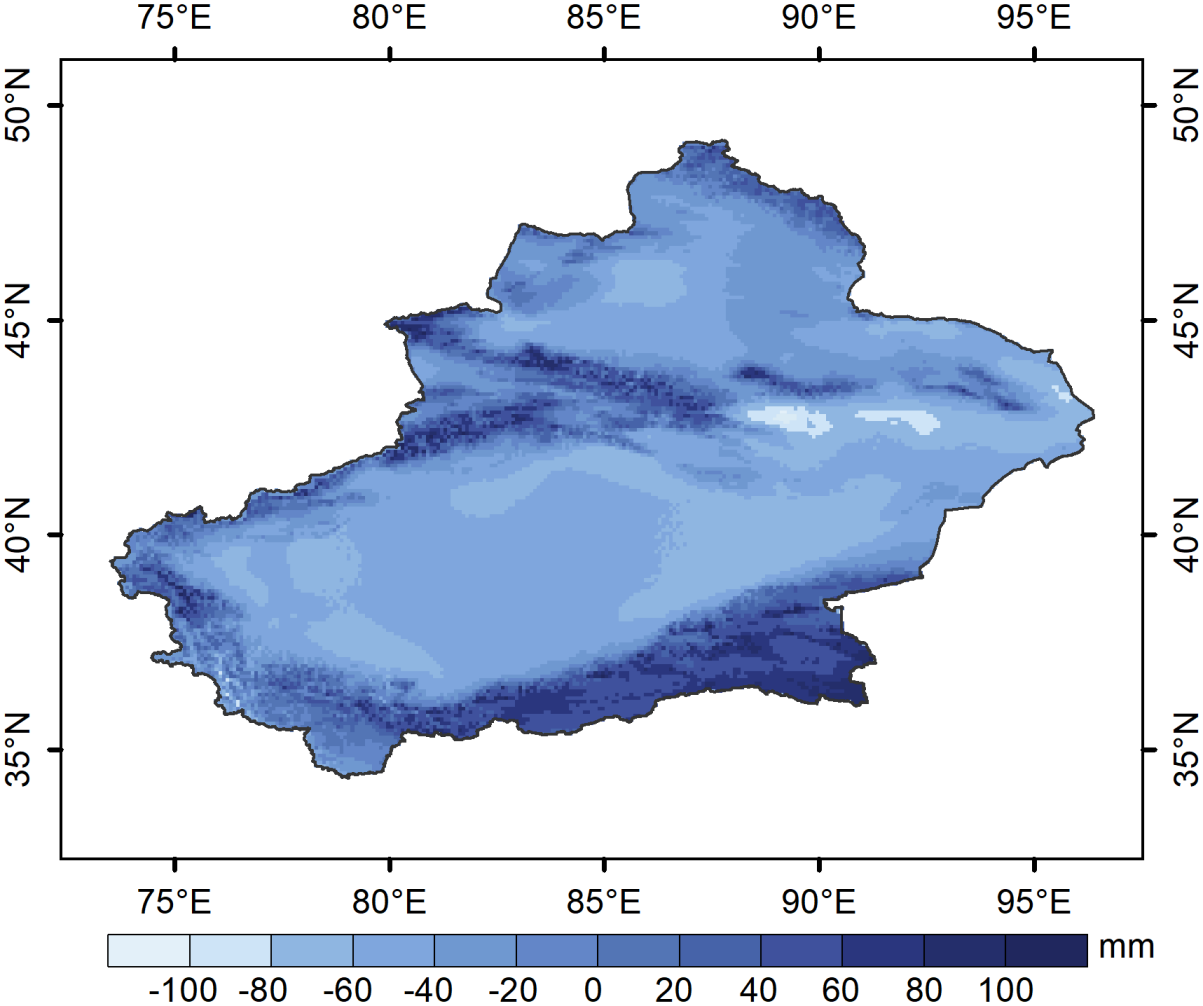
Mean values of growing season water balance in Xinjiang from 1982-2020.

In addition, the negative correlation between the SPEI-NDVI in January-June and August-October in the Altun Mountain grasslands was due to the change in atmospheric circulation on the Tibetan Plateau, while the third polar region changed the spatial pattern of its stock water bodies. The strengthening of westerly winds and weakening of the Indian monsoon led to an increase in precipitation in the northern (Kunlun and Altun Mountains) inland flow area and a decrease in precipitation in the southern outland flow area (Yao et al., 2022). In relatively humid regions, a negative SPEI during the grassland growing season (April-October) did not mean that vegetation was short of water, as the water balance was positive during periods of high precipitation (**Figure 13**). Therefore, dry and wet conditions during the growing season of grassland vegetation were not necessarily limiting factors, as seen from the SPEI-NDVI correlations for the southern and northern regions.

### 4.2 Heterogeneity of climate zones and grassland types in response to drought

The scale of vegetation response to drought varied depending on the climatic regions and vegetation type in which it was located (Zhang et al., 2017; Zhang and Zhang, 2019). Our study was only on grassland vegetation, so the scale of grassland response to drought varied mainly with the climatic regions in which grassland was located. Grasslands in semi-humid regions take longer to respond to moisture changes than do grasslands in arid and semiarid regions in our study. The SPEI in semi-humid regions is negative, but the water balance tends to be positive (Zhou et al., 2018), making grasslands in wetter areas less responsive to drought than those in drier regions. The results also suggest that grassland strategies for moisture changes in different regions may vary depending on the climate regions in which they are located. Grasslands are limited in water supply in different climatic regions (Cao et al., 2022; Liu et al., 2022). Grasslands in arid and semiarid regions responded more strongly to water deficits, possibly due to the negative average water balance during grassland vegetation growth (**Figure 13**), and the increased demand for water from grassland vegetation growth, leaves, and roots led to shorter response times to water deficits, as vegetation response time tended to be positively correlated with water balance in the near-zero degree range (Vicente-Serrano et al., 2013). Compared to arid and semiarid regions, the average water balance vegetation growth periods were positive in subhumid regions (**Figure 13**). Long-term positive water balance accumulation also boosted soil moisture and decreased the sensitivity of grassland development to short-term water shortage, allowing grassland to adapt to long-term water deficiency. The results were consistent with those of Fang et al. (2019b) in mainland China and on the Loess Plateau.

The reason for the highest probability of grassland NDVI declining in April in spring when the arid region is subjected to drought is that the natural factor that has a greater impact on grass in April in spring is the gradual increase in temperature, since precipitation in the dry area is consistently low throughout the growing season (Zhang et al., 2020). Drought in semiarid regions is most likely to cause grassland losses in August and September of summer and autumn, and the results were consistent with those of Yuan et al. (2022) in Central Asia (Yuan et al., 2022). This result is possibly due to the persistent high temperatures in Xinjiang at this time of year and the fact that semiarid regions are more dependent on precipitation than are semi-humid regions and appear to have a greater demand for soil moisture from iceberg meltwater (Zheng et al., 2021). In addition, spring warming leads to melting of alpine snow and ice and increased soil moisture, whereas at higher altitudes, the heat required for melting of snow and ice and evaporation of liquid water further leads to lower soil temperatures (Su et al., 2011). The microbial activity and root growth metabolic capacity of herbaceous plants are affected by lower soil temperatures (Ni and Li, 2019), which in turn reduces the water uptake capacity of grassland vegetation. In addition, high or low air humidity can cause stomatal closure of grasses, reduced transpiration and photosynthesis, inability of grasses to transport mineral nutrients well, and growth inhibition (Li et al., 2022).

**Figure 14:**
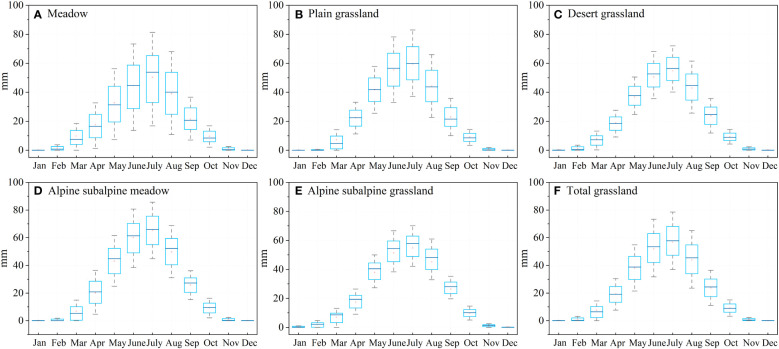
Seasonal variation in evapotranspiration in different grassland types: **(A)** meadows, **(B)** plains grasslands, **(C)** desert grasslands, **(D)** alpine subalpine meadows, **(E)** alpine subalpine grasslands, and **(F)** Total grasslands.

In our results, meadows were more sensitive to drought in April in the spring and less sensitive in the drier months of August and September. This result is due to the presence of rivers in the main distribution area of the meadow, as rivers can compensate for the water demand of the meadow during drought (**Figure 1B**). Desert grasslands are often water scarce and have evolved physiological mechanisms capable of adapting to water-scarce conditions, such as more efficient water storage systems and stronger roots, after a long period of survival and adaptation (Guo et al., 2012; Lei et al., 2020). The low sensitivity of desert grasslands to drought in the study results is consistent with previous regional and national studies (Huang et al., 2021; Bu et al., 2022). It is important to emphasize that seasonal changes in vegetation also affect evapotranspiration (Fu et al., 2023), and **Figure 14** shows the seasonal changes in evapotranspiration in response to different grassland types. In our study, all five grassland types showed the same trend, with July being the period of maximum evapotranspiration, differing from alpine subalpine meadows where evapotranspiration was greatest. In the drier month of August, the evapotranspiration of the meadow decreased instead, which also indicates that the meadow adopted appropriate strategies to prevent water loss during drought (Yue et al., 2019; Li et al., 2020). Therefore, more attention was given to plain grasslands and alpine subalpine meadows, which have a higher probability of water loss during drought.

### 4.3 Main influencing factors of drought stress in grassland

In general, temperature, precipitation and evapotranspiration are the main causes that directly control the exposure of vegetation to drought stress (Ashraf et al., 2022; Zhao et al., 2022). Xinjiang has long daylight hours and strong solar radiation (He et al., 2021). However, as the vegetation growth period changes, its main influencing factors subsequently change. In our study, the main influencing factor in April (spring) was temperature (73.24%), which was because most of the grasses in Xinjiang grow in higher-altitude areas and need sufficient temperature to melt snow and ice at the beginning of growth to provide the water and heat needed for grass growth (Zhang et al., 2019). The main influencing factors in August (summer) were temperature (48.10%) and evapotranspiration (44.72%), which was due to the low precipitation and persistent high temperature in summer, resulting in increased evapotranspiration and long-term water deficits in grasslands (Huang et al., 2017). The main influencing factor in September (autumn) was evapotranspiration (48.55%), which was because evapotranspiration became the main influencing factor in the late growth period when grass vigor decreased along with the ability to retain water (Du et al., 2015).

**Figure 15:**
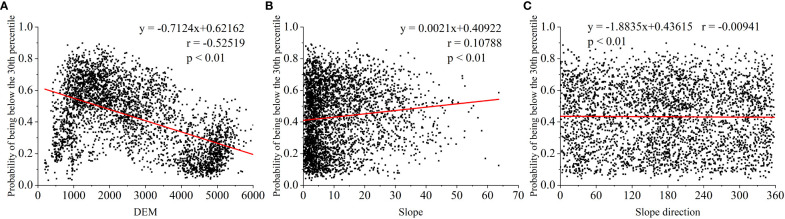
Correlation between elevation, slope and slope orientation and the probability of decline of grass-land NDVI to the lower percentiles under drought stress: **(A)** DEM, **(B)** slope and **(C)** slope direction (as an example, the probability of falling to the 30th percentile under severe drought stress in Au-gust, and the most severe drought was in summer).

In addition to climatic factors, other environmental factors (e.g., elevation, slope and aspect) can influence the drought patterns in a region (Kumari et al., 2020). Because elevation is a direct control factor affecting temperature, slope orientation affects solar exposure time and solar radiation (P. Zhang et al., 2022), both of which are important factors affecting vegetation drought (Yang et al., 2022). The only highly significant correlation (r = -0.52519) found in our study was between elevation and the probability of meadow decline to the 30th percentile of the land during severe drought, with a smaller effect of slope and slope orientation on meadow drought (**Figure 15**). This is because the NDVI representing grasslands in our study used lower resolution (1/12°) GIMMS data that do not capture the effects of slope and slope orientation on grassland drought (Chen et al., 2021).

Moreover, most of the grasslands in Xinjiang grow in cold and dry areas, and freezing and cold effects are also important factors affecting the growth of grasslands (Wang et al., 2022). This part of the grassland community is more homogeneous, and lower soil temperatures, moisture and nutrients under freezing and cold conditions limit the growth of grasslands, which in turn slow photosynthesis and carbon cycling (Rixen et al., 2022). In addition, there are limitations in our study; this study used GIMMS NDVI data with long time series but low resolution because it is more convincing to use a longer time when considering the effect of climate change on vegetation. The enhanced vegetation index (EVI) is also a better index for monitoring vegetation (Wu et al., 2022). Under the condition of better vegetation growth, the EVI can capture more vegetation changes than the NDVI, but it has limited application in mountainous areas (Kumari et al., 2021). Therefore, we prefer to use the EVI to monitor vegetation when it is not limited by the length of time and topographic conditions. In future studies, more accurate remote sensing images, such as Sentinel, Landsat and SPOT images or multisource remote sensing image fusion methods, should be used to monitor vegetation activity.

### 4.4 Grassland activities to overcome water deficits and surpluses

Grasslands under drought stress will adopt appropriate strategies to mitigate drought and maintain their own growth and development (Singh et al., 2020). The most important of these is the regulation of photosynthesis, which is manifested by increasing the efficiency of water use, using less water to complete the same degree of photosynthesis (Linderson et al., 2012), producing more antioxidants to protect chlorophyll, etc., to reduce their own oxidative damage (Baccari et al., 2020) and enhancing photosynthesis to obtain more energy to regulate growth and development. Regulation of stomatal conductance is also an important strategy used by grasses to overcome drought (BiBi et al., 2021). In the case of water deficit, the grass will reduce the degree and number of stomatal openings and thus transpiration and water loss, which at the same time will reduce the CO_2_ concentration and reduce the efficiency of photosynthesis, resulting in the limitation of grass growth (Nunes et al., 2020; Baca Cabrera et al., 2021; Israel et al., 2022). Conversely, in water surpluses, the degree and number of stomatal openings are increased, accelerating respiration and the rate of water discharge to avoid root hypoxia and decay as well as the accumulation of salts and toxins in the body (Mielke et al., 2003; Pociecha et al., 2008; Nasrullah et al., 2022).

## 5 Conclusion

This study combined correlation analysis and cointegration theory to quantify the drought stress vulnerability of grasslands in Xinjiang from a probabilistic perspective. The response time of grassland vegetation varied among climate zones, with grasslands in humid regions having longer response times during the growing season than those in semiarid regions. More severe grassland drought led to a higher likelihood that a grassland would experience significant vegetation declines and take appropriate adaptation strategies to mitigate drought after vegetation deterioration to a certain level. Compared with other months, August was the most vulnerable period for drought in the Xinjiang grasslands. The highest probability of drought vulnerability was found in semiarid grasslands, as well as in plains grasslands and alpine subalpine grasslands. In addition, the main influencing factors were temperature in April and August and evapotranspiration in September.

## Data availability statement

The original contributions presented in the study are included in the article/supplementary material. Further inquiries can be directed to the corresponding author.

## Author contributions

WH: Conceptualization, Methodology, Writing-Original Draft, Visualization. JG: Methodology, Writing-review & editing. YL: Formal analysis. XJ: Investigation, Data curation. LL: Validation. JL: Investigation. XM: Formal analysis. CL: Investigation. JZ: Writing - review & editing, Project administration. All authors contributed to the article and approved the submitted version.

## References

[B1] Ashraf M. Ullah K. Adnan S. (2022). Satellite based impact assessment of temperature and rainfall variability on drought indices in southern Pakistan. Int. J. Appl. Earth Obs. Geoinformation 108, 102726. doi: 10.1016/j.jag.2022.102726

[B2] Baca Cabrera J. C. Hirl R. T. Schäufele R. Macdonald A. Schnyder H. (2021). Stomatal conductance limited the CO2 response of grassland in the last century. BMC Biol. 19, 50. doi: 10.1186/s12915-021-00988-4 33757496 PMC7989024

[B3] Baccari S. Elloumi O. Chaari-Rkhis A. Fenollosa E. Morales M. Drira N.. (2020). Linking leaf water potential, photosynthesis and chlorophyll loss with mechanisms of photo- and antioxidant protection in juvenile olive trees subjected to severe drought. Front. Plant Sci. 11. doi: 10.3389/fpls.2020.614144 PMC7759475 33362839

[B4] Beer C. Reichstein M. Ciais P. Farquhar G. D. Papale D. (2007). Mean annual GPP of Europe derived from its water balance. Geophys. Res. Lett. 34, L05401. doi: 10.1029/2006gl029006

[B5] Bento V. A. Gouveia C. M. DaCamara C. C. Trigo I. F. (2018). A climatological assessment of drought impact on vegetation health index. Agric. For. Meteorol. 259, 286–295. doi: 10.1016/j.agrformet.2018.05.014

[B6] BiBi S. Ahmad M. S. A. Hameed M. (2021). Role of leaf micro-structural and topographical traits in ecological success of some arid zone grasses. Pak. J. Bot. 53 (2), 691–700. doi: 10.30848/PJB2021-2(43)

[B7] Bu L. Lai Q. Qing S. Bao Y. Liu X. Na Q.. (2022). Grassland biomass inversion based on a random forest algorithm and drought risk assessment. Remote Sens. 14, 5745. doi: 10.3390/rs14225745

[B8] Cao S. Zhang L. He Y. Zhang Y. Chen Y. Yao S.. (2022). Effects and contributions of meteorological drought on agricultural drought under different climatic zones and vegetation types in Northwest China. Sci. Total Environ. 821, 153270. doi: 10.1016/j.scitotenv.2022.153270 35085634

[B9] Castellaneta M. Rita A. Camarero J. J. Colangelo M. Ripullone F. (2022). Declines in canopy greenness and tree growth are caused by combined climate extremes during drought-induced dieback. Sci. Total Environ. 813, 152666. doi: 10.1016/j.scitotenv.2021.152666 34968613

[B10] Chen F. Liu Z. Zhong H. Wang S. (2021). Exploring the applicability and scaling effects of satellite-observed spring and autumn phenology in complex terrain regions using four different spatial resolution products. Remote Sens. 13, 4582. doi: 10.3390/rs13224582

[B11] Chen N. Zhang Y. Song C. Xu M. Zhang T. Li M.. (2022). The chained effects of earlier vegetation activities and summer droughts on ecosystem productivity on the Tibetan plateau. Agric. For. Meteorol. 321, 108975. doi: 10.1016/j.agrformet.2022.108975

[B12] Du J. Shu J. Yin J. Yuan X. Jiaerheng A. Xiong S.. (2015). Analysis on spatio-temporal trends and drivers in vegetation growth during recent decades in xinjiang, China. Int. J. Appl. Earth Obs. Geoinformation 38, 216–228. doi: 10.1016/j.jag.2015.01.006

[B13] Du Q. Sun Y. Guan Q. Pan N. Wang Q. Ma Y.. (2022). Vulnerability of grassland ecosystems to climate change in the qilian mountains, northwest China. J. Hydrol. 612, 128305. doi: 10.1016/j.jhydrol.2022.128305

[B14] Duan Y. Luo M. Guo X. Cai P. Li F. (2021). Study on the relationship between snowmelt runoff for different latitudes and vegetation growth based on an improved SWAT model in xinjiang, China. Sustainability 13, 1189. doi: 10.3390/su13031189

[B15] Dutta D. Kundu A. Patel N. R. Saha S. K. Siddiqui A. R. (2015). Assessment of agricultural drought in rajasthan (India) using remote sensing derived vegetation condition index (VCI) and standardized precipitation index (SPI). Egypt J. Remote Sens. Space Sci. 18, 53–63. doi: 10.1016/j.ejrs.2015.03.006

[B16] Estel S. Kuemmerle T. Alcántara C. Levers C. Prishchepov A. Hostert P. (2015). Mapping farmland abandonment and recultivation across Europe using MODIS NDVI time series. Remote Sens. Environ. 163, 312–325. doi: 10.1016/j.rse.2015.03.028

[B17] Fang W. Huang S. Huang G. Huang Q. Wang H. Wang L.. (2019a). Copulas-based risk analysis for inter-seasonal combinations of wet and dry conditions under a changing climate. Int. J. Climatol. 39, 2005–2021. doi: 10.1002/joc.5929

[B18] Fang W. Huang S. Huang Q. Huang G. Meng E. Luan J. (2018). Reference evapotranspiration forecasting based on local meteorological and global climate information screened by partial mutual information. J. Hydrol. 561, 764–779. doi: 10.1016/j.jhydrol.2018.04.038

[B19] Fang W. Huang S. Huang Q. Huang G. Wang H. Leng G.. (2019b). Probabilistic assess-ment of remote sensing-based terrestrial vegetation vulnerability to drought stress of the Loess Plateau in China. Remote Sens. Environ. 232, 111290. doi: 10.1016/j.rse.2019.111290

[B20] Fang W. Huang S. Huang Q. Huang G. Wang H. Leng G.. (2019c). Bivariate probabilistic quantification of drought impacts on terrestrial vegetation dynamics in mainland China. J. Hydrol. 577, 123980. doi: 10.1016/j.jhydrol.2019.123980

[B21] Fu J. Wang W. Liu B. Lu Y. Xing W. Cao M.. (2023). Seasonal divergence of evapotranspiration sensitivity to vegetation changes – a proportionality-hypothesis-based analytical solution. J. Hydrol. 617, 129055. doi: 10.1016/j.jhydrol.2022.129055

[B22] Gouveia C. M. Trigo R. M. Beguería S. Vicente-Serrano S. M. (2017). Drought impacts on vegetation activity in the Mediterranean region: An assessment using remote sensing data and multi-scale drought indicators. Glob. Planet. Change 151, 15–27. doi: 10.1016/j.gloplacha.2016.06.011

[B23] Grimaldi S. Petroselli A. Salvadori G. De Michele C. (2016). Catchment compatibility *via* copulas: A non-parametric study of the dependence structures of hydrological responses. Adv. Water Resour. 90, 116–133. doi: 10.1016/j.advwatres.2016.02.003

[B24] Guan J. Yao J. Li M. Zheng J. (2021). Assessing the spatiotemporal evolution of anthropogenic impacts on remotely sensed vegetation dynamics in xinjiang, China. Remote Sens. 13, 4651. doi: 10.3390/rs13224651

[B25] Guli J. Liang S. Yi Q. Liu J. (2015). Vegetation dynamics and responses to recent climate change in xinjiang using leaf area index as an indicator. Ecol. Indic. 58, 64–76. doi: 10.1016/j.ecolind.2015.05.036

[B26] Guo Q. Hu Z. Li S. Li X. Sun X. Yu G. (2012). Spatial variations in aboveground net primary productivity along a climate gradient in Eurasian temperate grassland: effects of mean annual precipitation and its seasonal distribution. Glob. Change Biol. 18, 3624–3631. doi: 10.1111/gcb.12010

[B27] He P. Sun Z. Han Z. Dong Y. Liu H. Meng X.. (2021). Dynamic characteristics and driving factors of vegetation greenness under changing environments in xinjiang, China. Environ. Sci. pollut. Res. 28, 42516–42532. doi: 10.1007/s11356-021-13721-z 33813700

[B28] HOLBEN B. N. (1986). Characteristics of maximum-value composite images from temporal AVHRR data. Int. J. Remote Sens. 7, 1417–1434. doi: 10.1080/01431168608948945

[B29] Huang X. Luo G. Lv N. (2017). Spatio-temporal patterns of grassland evapotranspiration and water use efficiency in arid areas. Ecol. Res. 32, 523–535. doi: 10.1007/s11284-017-1463-2

[B30] Huang W. Wang W. Cao M. Fu G. Xia J. Wang Z.. (2021). Local climate and biodiversity affect the stability of china’s grasslands in response to drought. Sci. Total Environ. 768, 145482. doi: 10.1016/j.scitotenv.2021.145482 33736341

[B31] Israel W. K. Watson-Lazowski A. Chen Z.-H. Ghannoum O. (2022). High intrinsic water use efficiency is underpinned by high stomatal aperture and guard cell potassium flux in C3 and C4 grasses grown at glacial CO2 and low light. J. Exp. Bot. 73, 1546–1565. doi: 10.1093/jxb/erab477 34718533

[B32] Jackson R. D. Kustas W. P. Choudhury B. J. (1988). A reexamination of the crop water stress index. Irrig. Sci. 9, 309–317. doi: 10.1007/BF00296705

[B33] Ji L. Peters A. J. (2003). Assessing vegetation response to drought in the northern great plains using vegetation and drought indices. Remote Sens. Environ. 87, 85–98. doi: 10.1016/S0034-4257(03)00174-3

[B34] Kowalski K. Okujeni A. Brell M. Hostert P. (2022). Quantifying drought effects in central European grasslands through regression-based unmixing of intra-annual sentinel-2 time series. Remote Sens. Environ. 268, 112781. doi: 10.1016/j.rse.2021.112781

[B35] Kumari N. Saco P. M. Rodriguez J. F. Johnstone S. A. Srivastava A. Chun K. P.. (2020). The grass is not always greener on the other side: Seasonal reversal of vegetation greenness in aspect-driven semiarid ecosystems. Geophys. Res. Lett. 47, e2020GL088918. doi: 10.1029/2020GL088918

[B36] Kumari N. Srivastava A. Dumka U. C. (2021). A long-term spatiotemporal analysis of vegetation greenness over the Himalayan region using Google earth engine. Climate 9, 109. doi: 10.3390/cli9070109

[B37] Lei T. Feng J. Lv J. Wang J. Song H. Song W.. (2020). Net primary productivity loss under different drought levels in different grassland ecosystems. J. Environ. Manage. 274, 111144. doi: 10.1016/j.jenvman.2020.111144 32798851

[B38] Li H. W. Li Y. P. Huang G. H. Sun J. (2021). Quantifying effects of compound dry-hot extremes on vegetation in xinjiang (China) using a vine-copula conditional probability model. Agric. For. Meteorol. 311, 108658. doi: 10.1016/j.agrformet.2021.108658

[B39] Li S. Liu F. (2022). Vapour pressure deficit and endogenous ABA level modulate stomatal responses of tomato plants to soil water deficit. Environ. Exp. Bot. 199, 104889. doi: 10.1016/j.envexpbot.2022.104889

[B40] Li J. Peng S. Li Z. (2017). Detecting and attributing vegetation changes on china’s loess plateau. Agric. For. Meteorol. 247, 260–270. doi: 10.1016/j.agrformet.2017.08.005

[B41] Li L. Qian R. Liu W. Wang W. Biederman J. A. Zhang B.. (2022). Drought timing influences the sensitivity of a semiarid grassland to drought. Geoderma 412, 115714. doi: 10.1016/j.geoderma.2022.115714

[B42] Li L. Song X. Xia L. Fu N. Feng D. Li H.. (2020). Modelling the effects of climate change on transpiration and evaporation in natural and constructed grasslands in the semi-arid loess plateau, China. Agric. Ecosyst. Environ. 302, 107077. doi: 10.1016/j.agee.2020.107077

[B43] Li J. Xi M. Pan Z. Liu Z. He Z. Qin F. (2022). Response of NDVI and SIF to meteorological drought in the yellow river basin from 2001 to 2020. Water 14, 2978. doi: 10.3390/w14192978

[B44] Linderson M.-L. Mikkelsen T. Ibrom A. Lindroth A. Ro-Poulsen H. Pilegaard K. (2012). Up-scaling of water use efficiency from leaf to canopy as based on leaf gas exchange relationships and the modeled in-canopy light distribution. Agric. For. Meteorol. 152, 201–211. doi: 10.1016/j.agrformet.2011.09.019

[B45] Liu Y. Li L. Chen X. Zhang R. Yang J. (2018). Temporal-spatial variations and influencing factors of vegetation cover in xinjiang from 1982 to 2013 based on GIMMS-NDVI3g. Glob. Planet. Change 169, 145–155. doi: 10.1016/j.gloplacha.2018.06.005

[B46] Liu Z. Li C. Zhou P. Chen X. (2016). A probabilistic assessment of the likelihood of vegetation drought under varying climate conditions across China. Sci. Rep. 6, 35105. doi: 10.1038/srep35105 27713530 PMC5054395

[B47] Liu X. Liu C. Fan B. Li L. Tan B. Jin Z.. (2022). Spatial responses of ecosystem water-use efficiency to hydrothermal and vegetative gradients in alpine grassland ecosystem in drylands. Ecol. Indic. 141, 109064. doi: 10.1016/j.ecolind.2022.109064

[B48] Liu Y. Yang Y. Wang Q. Du X. Li J. Gang C.. (2019). Evaluating the responses of net primary productivity and carbon use efficiency of global grassland to climate variability along an aridity gradient. Sci. Total Environ. 652, 671–682. doi: 10.1016/j.scitotenv.2018.10.295 30380475

[B49] Lu Y. Cai H. Jiang T. Sun S. Wang Y. Zhao J.. (2019). Assessment of global drought propensity and its impacts on agricultural water use in future climate scenarios. Agric. For. Meteorol. 278, 107623. doi: 10.1016/j.agrformet.2019.107623

[B50] Mazdiyasni O. AghaKouchak A. Davis S. J. Madadgar S. Mehran A. Ragno E.. (2017). Increasing probability of mortality during Indian heat waves. Sci. Adv. 3, e1700066. doi: 10.1126/sciadv.1700066 28630921 PMC5462497

[B51] McKee T. B. Doesken N. J. Kleist J. (1993). “The relationship of drought frequency and duration to time scales,” in Eighth Conference on Applied Climatology, Meteorological Society, Anaheim, California, America. pp. 179–183. Available at: http://ccc.atmos.colostate.edu/relationshipofdroughtfrequency.pdf.

[B52] Mielke M. S. de Almeida A.-A. F. Gomes F. P. Aguilar M. A. G. Mangabeira P. A. O. (2003). Leaf gas exchange, chlorophyll fluorescence and growth responses of genipa americana seedlings to soil flooding. Environ. Exp. Bot. 50, 221–231. doi: 10.1016/S0098-8472(03)00036-4

[B53] Mina M. Martin-Benito D. Bugmann H. Cailleret M. (2016). Forward modeling of tree-ring width improves simulation of forest growth responses to drought. Agric. For. Meteorol. 221, 13–33. doi: 10.1016/j.agrformet.2016.02.005

[B54] Mohammat A. Wang X. Xu X. Peng L. Yang Y. Zhang X.. (2013). Drought and spring cooling induced recent decrease in vegetation growth in inner Asia. Agric. For. Meteorol. Special Issue:Drought Inner Asia 178–179, 21–30. doi: 10.1016/j.agrformet.2012.09.014

[B55] Nalbantis I. Tsakiris G. (2009). Assessment of hydrological drought revisited. Water Resour. Manage. 23, 881–897. doi: 10.1007/s11269-008-9305-1

[B56] Nasrullah A. Umar M. Sun L. Naeem M. Yasmin H. Khan N. (2022). Flooding tolerance in plants: from physiological and molecular perspectives. Braz. J. Bot. 45, 1161–1176. doi: 10.1007/s40415-022-00841-0

[B57] Neuner G. Huber B. Plangger A. Pohlin J.-M. Walde J. (2020). Low temperatures at higher elevations require plants to exhibit increased freezing resistance throughout the summer months. Environ. Exp. Bot. 169, 103882. doi: 10.1016/j.envexpbot.2019.103882

[B58] Ni M. Li S. (2019). Biodegradability of riverine dissolved organic carbon in a dry-hot valley region: Initial trophic controls and variations in chemical composition. J. Hydrol. 574, 430–435. doi: 10.1016/j.jhydrol.2019.04.069

[B59] Nunes T. D. G. Zhang D. Raissig M. T. (2020). Form, development and function of grass stomata. Plant J. 101, 780–799. doi: 10.1111/tpj.14552 31571301

[B60] Palmer W. C. (1965). Meteorological drought (Weather Bureau: U.S. Department of Commerce).

[B61] Palmer W. C. (1968). Keeping track of crop moisture conditions, nationwide: The new crop moisture index. Weatherwise 21, 156–161. doi: 10.1080/00431672.1968.9932814

[B62] Piao S. Zhang X. Chen A. Liu Q. Lian X. Wang X.. (2019). The impacts of climate extremes on the terrestrial carbon cycle: A review. Sci. China Earth Sci. 62, 1551–1563. doi: 10.1007/s11430-018-9363-5

[B63] Pociecha E. Kościelniak J. Filek W. (2008). Effects of root flooding and stage of development on the growth and photosynthesis of field bean (Vicia faba l. minor). Acta Physiol. Plant 30, 529–535. doi: 10.1007/s11738-008-0151-9

[B64] Reichstein M. Bahn M. Ciais P. Frank D. Mahecha M. D. Seneviratne S. I.. (2013). Climate extremes and the carbon cycle. Nature 500, 287–295. doi: 10.1038/nature12350 23955228

[B65] Rixen C. Høye T. T. Macek P. Aerts R. Alatalo J. M. Anderson J. T.. (2022). Winters are changing: snow effects on Arctic and alpine tundra ecosystems. Arct. Sci. 8, 572–608. doi: 10.1139/as-2020-0058

[B66] Shukla S. Wood A. W. (2008). Use of a standardized runoff index for characterizing hydrologic drought. Geophys. Res. Lett. 35, L02405. doi: 10.1029/2007GL032487

[B67] Singh C. Wang-Erlandsson L. Fetzer I. Rockström J. van der Ent R. (2020). Rootzone storage capacity reveals drought coping strategies along rainforest-savanna transitions. Environ. Res. Lett. 15, 124021. doi: 10.1088/1748-9326/abc377

[B68] Su Z. Wen J. Dente L. van der Velde R. Wang L. Ma Y.. (2011). The Tibetan plateau observatory of plateau scale soil moisture and soil temperature (Tibet-obs) for quantifying uncertainties in coarse resolution satellite and model products. Hydrol. Earth Syst. Sci. 15, 2303–2316. doi: 10.5194/hess-15-2303-2011

[B69] Thornthwaite C. W. (1948). An approach toward a rational classification of climate. Geogr. Rev. 38 (1), 55–94.

[B70] Vicente-Serrano S. M. Beguería S. López-Moreno J. I. (2010). A multiscalar drought index sensitive to global warming: the standardized precipitation evapotranspiration index. J. Clim. 23, 1696–1718. doi: 10.1175/2009JCLI2909.1

[B71] Vicente-Serrano S. M. Gouveia C. Camarero J. J. Beguería S. Trigo R. López-Moreno J. I.. (2013). Response of vegetation to drought time-scales across global land biomes. Proc. Natl. Acad. Sci. 110, 52–57. doi: 10.1073/pnas.1207068110 23248309 PMC3538253

[B72] Wang Y. Fu B. Liu Y. Li Y. Feng X. Wang S. (2021). Response of vegetation to drought in the Tibetan plateau: Elevation differentiation and the dominant factors. Agric. For. Meteorol. 306, 108468. doi: 10.1016/j.agrformet.2021.108468

[B73] Wang C. Wang A. Guo D. Li H. Zang S. (2022). Off-peak NDVI correction to reconstruct landsat time series for post-fire recovery in high-latitude forests. Int. J. Appl. Earth Obs. Geoinformation 107, 102704. doi: 10.1016/j.jag.2022.102704

[B74] Wang T. Yang D. Zheng G. Shi R. (2022). Possible negative effects of earlier thaw onset and longer thaw duration on vegetation greenness over the Tibetan plateau. Agric. For. Meteorol. 326, 109192. doi: 10.1016/j.agrformet.2022.109192

[B75] Wellstein C. Poschlod P. Gohlke A. Chelli S. Campetella G. Rosbakh S.. (2017). Effects of extreme drought on specific leaf area of grassland species: A meta-analysis of experimental studies in temperate and sub-Mediterranean systems. Glob. Change Biol. 23, 2473–2481. doi: 10.1111/gcb.13662 28208238

[B76] Wen L. Yang X. Saintilan N. (2012). Local climate determines the NDVI-based primary productivity and flooding creates heterogeneity in semi-arid floodplain ecosystem. Ecol. Model. 242, 116–126. doi: 10.1016/j.ecolmodel.2012.05.018

[B77] Wilcox K. R. Koerner S. E. Hoover D. L. Borkenhagen A. K. Burkepile D. E. Collins S. L.. (2020). Rapid recovery of ecosystem function following extreme drought in a south African savanna grassland. Ecology 101, e02983. doi: 10.1002/ecy.2983 31960960

[B78] Won J. Seo J. Kim S. (2022). A copula model integrating atmospheric moisture demand and supply for vegetation vulnerability mapping. Sci. Total Environ. 812, 151464. doi: 10.1016/j.scitotenv.2021.151464 34742982

[B79] Won J. Seo J. Lee J. Lee O. Kim S. (2021). Vegetation drought vulnerability mapping using a copula model of vegetation index and meteorological drought index. Remote Sens. 13, 5103. doi: 10.3390/rs13245103

[B80] Wu G. Chen J. Kim J.-S. Gu L. Lee J.-H. Zhang L. (2022). Impacts of climate change on global meteorological multi-year droughts using the last millennium simulation as a baseline. J. Hydrol. 610, 127937. doi: 10.1016/j.jhydrol.2022.127937

[B81] Wu M. Manzoni S. Vico G. Bastos A. de Vries F. T. Messori G. (2022). Drought legacy in Sub-seasonal vegetation state and sensitivity to climate over the northern hemisphere. Geophys. Res. Lett. 49, e2022GL098700. doi: 10.1029/2022GL098700

[B82] Xu H. Wang X. Zhang X. (2016). Decreased vegetation growth in response to summer drought in central Asia from 2000 to 2012. Int. J. Appl. Earth Obs. Geoinformation 52, 390–402. doi: 10.1016/j.jag.2016.07.010

[B83] Xu Y. Yang J. Chen Y. (2016). NDVI-based vegetation responses to climate change in an arid area of China. Theor. Appl. Climatol. 126, 213–222. doi: 10.1007/s00704-015-1572-1

[B84] Xun L. Zhang J. Yao F. Cao D. (2022). Improved identification of cotton cultivated areas by applying instance-based transfer learning on the time series of MODIS NDVI. CATENA 213, 106130. doi: 10.1016/j.catena.2022.106130

[B85] Yang F. Duan X. Guo Q. Lu S. Hsu K. (2022). The spatiotemporal variations and propagation of droughts in plateau mountains of China. Sci. Total Environ. 805, 150257. doi: 10.1016/j.scitotenv.2021.150257 34536870

[B86] Yao T. Bolch T. Chen D. Gao J. Immerzeel W. Piao S.. (2022). The imbalance of the Asian water tower. Nat. Rev. Earth Environ. 3, 618–632. doi: 10.1038/s43017-022-00299-4

[B87] Yao J. Chen Y. Zhao Y. Guan X. Mao W. Yang L. (2020). Climatic and associated atmospheric water cycle changes over the xinjiang, china. J. Hydrol 585, 124823. doi: 10.1016/j.jhydrol.2020.124823

[B88] Yao Y. Fu B. Liu Y. Li Y. Wang S. Zhan T.. (2022). Evaluation of ecosystem resilience to drought based on drought intensity and recovery time. Agric. For. Meteorol. 314, 108809. doi: 10.1016/j.agrformet.2022.108809

[B89] Yao J. Hu W. Chen Y. Huo W. Zhao Y. Mao W.. (2019). Hydro-climatic changes and their impacts on vegetation in xinjiang, central Asia. Sci. Total Environ. 660, 724–732. doi: 10.1016/j.scitotenv.2019.01.084 30743958

[B90] Yao J. Zhao Y. Chen Y. Yu X. Zhang R. (2018). Multi-scale assessments of droughts: A case study in xinjiang, China. Sci. Total Environ. 630, 444–452. doi: 10.1016/j.scitotenv.2018.02.200 29486438

[B91] Yerdelen C. Abdelkader M. Eris E. (2021). Assessment of drought in SPI series using continuous wavelet analysis for gediz basin, Turkey. Atmospheric Res. 260, 105687. doi: 10.1016/j.atmosres.2021.105687

[B92] Yu K. Xiong L. Li P. Li Z. Zhang X. Sun Q. (2018). Analyzing the impacts of climatic and physiographic factors on low flow distributions. Water Resour. Manage. 32, 881–896. doi: 10.1007/s11269-017-1844-x

[B93] Yuan Y. Bao A. Jiang P. Hamdi R. Termonia P. De Maeyer P.. (2022). Probabilistic assessment of vegetation vulnerability to drought stress in central Asia. J. Environ. Manage. 310, 114504. doi: 10.1016/j.jenvman.2022.114504 35189553

[B94] Yue P. Zhang Q. Zhang L. Li H. Yang Y. Zeng J.. (2019). Long-term variations in energy partitioning and evapotranspiration in a semiarid grassland in the loess plateau of China. Agric. For. Meteorol. 278, 107671. doi: 10.1016/j.agrformet.2019.107671

[B95] Zeng N. Niu Z. Li P. Zhu X. Ren X. (2022). Resistance of grassland productivity to hydroclimatic changes in the Tibetan plateau. Ecol. Indic. 143, 109351. doi: 10.1016/j.ecolind.2022.109351

[B96] Zhang R. Guo J. Liang T. Feng Q. (2019). Grassland vegetation phenological variations and responses to climate change in the xinjiang region, China. Quat. Int. 513, 56–65. doi: 10.1016/j.quaint.2019.03.010

[B97] Zhang P. Jiao L. Wei M. Wu X. Du D. Xue R. (2022). Drought timing and severity affect radial growth of picea crassifolia at different elevations in the western qilian mountains. Int. J. Biometeorol. 66, 2449–2462. doi: 10.1007/s00484-022-02368-1 36201038

[B98] Zhang Z. Ju W. Zhou Y. Li X. (2022). Revisiting the cumulative effects of drought on global gross primary productivity based on new long-term series data, (1982–2018). Glob. Change Biol. 28, 3620–3635. doi: 10.1111/gcb.16178 35343026

[B99] Zhang Q. Kong D. Singh V. P. Shi P. (2017). Response of vegetation to different time-scales drought across China: Spatiotemporal patterns, causes and implications. Glob. Planet. Change 152, 1–11. doi: 10.1016/j.gloplacha.2017.02.008

[B100] Zhang N. Liu C. (2014). Simulated water fluxes during the growing season in semiarid grassland ecosystems under severe drought conditions. J. Hydrol. 512, 69–86. doi: 10.1016/j.jhydrol.2014.02.056

[B101] Zhang F. Wang C. Wang Z.-H. (2020). Response of natural vegetation to climate in dryland ecosystems: A comparative study between xinjiang and Arizona. Remote Sens. 12, 3567. doi: 10.3390/rs12213567

[B102] Zhang X. Zhang B. (2019). The responses of natural vegetation dynamics to drought during the growing season across China. J. Hydrol. 574, 706–714. doi: 10.1016/j.jhydrol.2019.04.084

[B103] Zhao J. Huang S. Huang Q. Wang H. Leng G. Peng J.. (2019). Copula-based abrupt variations detection in the relationship of seasonal vegetation-climate in the jing river basin, China. Remote Sens. 11, 1628. doi: 10.3390/rs11131628

[B104] Zhao M. Liu Y. Konings A. G. (2022). Evapotranspiration frequently increases during droughts. Nat. Clim. Change 12, 1024–1030. doi: 10.1038/s41558-022-01505-3

[B105] Zhao A. Yu Q. Feng L. Zhang A. Pei T. (2020). Evaluating the cumulative and time-lag effects of drought on grassland vegetation: A case study in the Chinese loess plateau. J. Environ. Manage. 261, 110214. doi: 10.1016/j.jenvman.2020.110214 32148284

[B106] Zhao A. Zhang A. Cao S. Liu X. Liu J. Cheng D. (2018). Responses of vegetation productivity to multi-scale drought in loess plateau, China. Catena 163, 165–171. doi: 10.1016/j.catena.2017.12.016

[B107] Zheng S. Zhang B. Peng D. Yu L. Lin B. Pan Y.. (2021). The trend towards a warmer and wetter climate observed in arid and semi-arid areas of northwest China from 1959 to 2019. Environ. Res. Commun. 3, 115011. doi: 10.1088/2515-7620/ac39f7

[B108] Zhong S. Sun Z. Di L. (2021). Characteristics of vegetation response to drought in the CONUS based on long-term remote sensing and meteorological data. Ecol. Indic. 127, 107767. doi: 10.1016/j.ecolind.2021.107767

[B109] Zhou X. Huang G. Wang X. Fan Y. Cheng G. (2018). A coupled dynamical-copula downscaling approach for temperature projections over the Canadian prairies. Clim. Dyn. 51, 2413–2431. doi: 10.1007/s00382-017-4020-3

[B110] Zhou Q. Luo Y. Zhou X. Cai M. Zhao C. (2018). Response of vegetation to water balance conditions at different time scales across the karst area of southwestern China–a remote sensing approach. Sci. Total Environ. 645, 460–470. doi: 10.1016/j.scitotenv.2018.07.148 30029121

